# Cryptic diets of forage fish: jellyfish consumption observed in the Celtic Sea and western English Channel

**DOI:** 10.1111/jfb.13926

**Published:** 2019-03-12

**Authors:** Philip D. Lamb, Ewan Hunter, John K. Pinnegar, Jeroen van der Kooij, Simon Creer, Martin I. Taylor

**Affiliations:** ^1^ School of Biological Sciences University of East Anglia Norfolk UK; ^2^ School of Environmental Sciences University of East Anglia Norfolk UK; ^3^ Cefas, Lowestoft Suffolk UK; ^4^ School of Biological Sciences Bangor University Bangor UK

**Keywords:** *16s* mtDNA, Celtic Sea, diet, English Channel, gelatinous zooplankton, molecular gut‐content analysis

## Abstract

To establish if fishes’ consumption of jellyfish changes through the year, we conducted a molecular gut‐content assessment on opportunistically sampled species from the Celtic Sea in October and compared these with samples previously collected in February and March from the Irish Sea. Mackerel *Scomber scombrus* were found to feed on hydrozoan jellyfish relatively frequently in autumn, with rare consumption also detected in sardine *Sardina pilchardus* and sprat *Sprattus sprattus*. By October, moon jellyfish *Aurelia aurita* appeared to have escaped predation, potentially through somatic growth and the development of stinging tentacles. This is in contrast with sampling in February and March where *A. aurita* ephyrae were heavily preyed upon. No significant change in predation rate was observed in *S. sprattus*, but jellyfish predation by *S. scombrus* feeding in autumn was significantly higher than that seen during winter. This increase in consumption appears to be driven by the consumption of different, smaller jellyfish species than were targeted during the winter.

## 1 INTRODUCTION

Fisheries in the Irish Sea are important for the regional economy: in 2016 the UK‐based fleet landed 36,600 t worth £57.8 million (Richardson *et al.*, 2017), while the Irish fleet caught a further 11,253 t (CSO, 2018). However, Irish Sea fisheries are facing challenges from increasingly abundant scyphomedusae jellyfish (hereafter referred to as jellyfish, unless stated otherwise; Lynam *et al.*, 2011). Jellyfish blooms (mass aggregations of jellyfish in a localised area) in other regions have caused economic losses to fisheries by bursting fishing nets, contaminating catches, reducing the abundance of fish by competing for the same resources and killing fish through irritation of gills with their stinging tentacles (Richardson *et al.*, 2009). Fish farms can also suffer damage and mass mortality from jellyfish blooms (Doyle *et al.*, 2008). Preventing jellyfish blooms from affecting human enterprise has been difficult and many direct interventions have been ineffective (Richardson *et al.*, 2009).

The significance of jellyfish in marine food webs has become clear through the application of stable‐isotope analysis (Cardona *et al.*, 2012; Utne‐Palm *et al.*, 2010), stationary underwater cameras (Sweetman & Chapman, 2011), remote operated vehicles (Hoving & Haddock, 2017) and acoustic surveys (Utne‐Palm *et al.*, 2010). Commercially‐important fish species such as herring *Clupea harengus* L. 1758 and whiting *Merlangius merlangus* L. 1758 were shown to consume jellyfish in the Irish Sea using a molecular assay (Lamb *et al.*, 2017). However, the observed scyphomedusae consumption occurred when jellyfish in the Irish Sea were juvenile and lacked the size or defensive structures to deter predation; it remains unknown if they are consumed throughout the year or used as a seasonal resource.

Complex and dynamic interspecific relationships are common in marine ecosystems: assuming unchanging predation throughout the year is likely to misrepresent a species’ trophic role. For example, *C. harengus* are known to limit cod *Gadus morhua* L. 1758 recruitment by feeding on juvenile *G. morhua* when they are part of the ichthyoplankton (Koster & Mollmann, 1996). However, upon maturation, *G. morhua* feed on small *C. harengus* (Bailey & Batty, 1984), reversing the interspecific relationship. A dynamic relationship like this may be present in jellyfish as they have a complex life cycle featuring multiple, functionally different life stages (Lucas, 2001). During February and March, consumption of jellyfish was probably targeting ephyrae (a juvenile form of jellyfish) (Lamb *et al.*, 2017). Ephyrae are just a few millimetres in diameter and often lack the stinging tentacles seen in mature jellyfish as these can take several weeks to develop once they join the plankton community (Holst, 2012).

Although all Irish Sea and Celtic Sea jellyfish ephyrae measure a few millimetres in diameter, there is considerable variation in size and stinging ability by maturation (Holst, 2012). Mauve stinger jellyfish *Pelagia noctiluca* (Forsskål, 1775) remain small, with a mean ± SD diameter of 4.5 (± 1.2) cm, although large individuals can reach 12 cm (Bastian *et al.*, 2011). Other common species are known to grow larger: *Aurelia aurita* L. 1758 can reach 25 cm diameter (Omori *et al.*, 1995), while barrel jellyfish bells *Rhizostoma pulmo* (Macri, 1778) are known to approach 1 m in diameter (Russell, 1970). Large predators such as leatherback turtles *Dermochelys coriacea* (Vandelli, 1761) feed on whole medusa (Heaslip *et al.*, 2012; Houghton *et al.*, 2006), but it remains to be seen if the pelagic fish species identified previously as jellyfish consumers in the Irish Sea (Lamb *et al.*, 2017) maintain this trophic relationship throughout the year. It is plausible that large size of jellyfish relative to the predatory fish and the development of stinging tentacles may limit predation. However, other predatory fish species have been observed biting and consuming, parts of jellyfish despite these structures *(*Milisenda *et al.*, 2014) so jellyfish may yet be viable prey.

Here, as a first step towards understanding the contribution of jellyfish in supporting fisheries, predation of mature jellyfish is characterised with the aim of testing whether jellyfish are consumed by commercially exploited fish species throughout the year.

## 2 MATERIAL AND METHODS

### 2.1 Sampling in 2015

**Figure 1:**
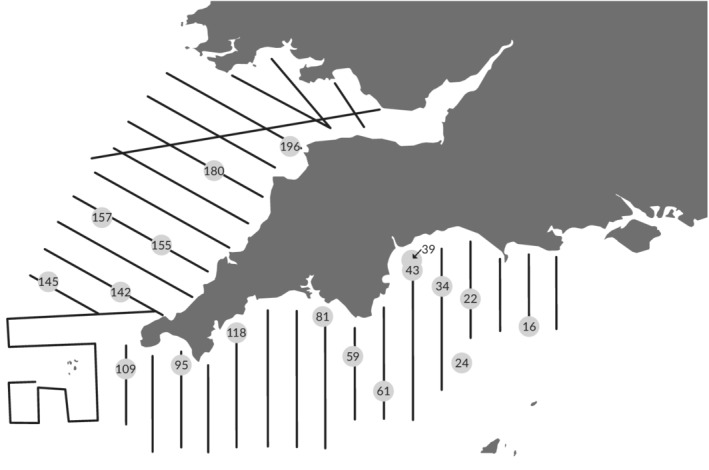
Acoustic survey grid (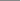) for the 2015 samples collected (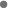) from the western English Channel and Celtic Sea. Sampling location numbers denote sampling station identity. Diagram adapted with permission from ICES (2016)

Samples were collected aboard the R.V. *Cefas Endeavour* as part of the PELTIC 15 research survey. Collection permits were not required, with all samples being caught and processed following Cefas guidelines (Cefas, 2017). Full details on the PELTIC 15 survey can be found in Appendix 5 of ICES WGIPS report (ICES, 2016). Briefly, between 05 and 20 October 2015 acoustic data acquisition and plankton sampling were undertaken along (Figure 1). A 20 × 40 m vdK herring trawl using KT nets was deployed opportunistically at 18 locations when fish schools were observed in the echograms (Figure 1). Upon retrieval of fish, they were identified to species level (Table 1), measured, weighed, and had their stomachs removed and frozen on‐board. Scalpels and gloves were changed and cutting boards cleaned using fresh water between species dissection. If jellyfish were found in the haul, they were identified to species level and a small sample of bell tissue was preserved in 100% ethanol.

**Table 1:** Samples which were tested for jellyfish consumption using molecular gut content analysis from PELTIC 2015 cruise

Common name	Scientific name	Sample size (*n*)	Samples containing jellyfish DNA
European anchovy	*Engraulis encrasicolus*	20	0
Horse mackerel	*Trachurus trachurus*	77	0
John dory	*Zeus faber*	5	0
Lesser spotted dogfish	*Scyliorhinus canicula*	1	0
Mackerel	*Scomber scombrus*	95	22
Sardine	*Sardina pilchardus*	70	1
Red gurnard	*Chelidonichthys cuculus*	5	0
Saury pike	*Scomberesox saurus*	5	0
European bass	*Dicentrarchus labrax*	4	0
Sprat	*Sprattus sprattus*	90	3
Whiting	*Merlangius merlangus*	3	0

Additional jellyfish samples were obtained from plankton sampling at night when the ship was stationary at designated samples points (ICES, 2016) using ring‐nets equipped with a General Oceanics 2030C mechamical flowmeter (http://www.genealoceanic.com) with either an 80 or 270 μm mesh as describted by Pitois *et al.*, (2016). Bell tissue was preserved in 100% ethanol. *n.b.,* This sampling was not jellyfish population sampling, rather a method for identifying jellyfish species present in the water column during the survey.

### 2.2 DNA extraction

Stomachs were thawed and contents dissected on a separate disposable paper towel and using flamed scissors, scalpel and forceps to prevent contamination. DNA was extracted using a salt extraction technique (Aljanabi & Martinez, 1997): Stomach contents were macerated and a small volume (*c*. 1–8 mm^3^) was placed in 300 μl digestion buffer (30 mM Tris–HCl ph 8.0, 10 mM EDTA, 1% sodium dodecyl sulphate (SDS), with 10 μl Proteinase‐K (Qiagen; http://www.qiagen.com)) in a 1.5 ml Eppendorf tube, incubated overnight at 55**°**C. One hundred microlitre of 5 M NaCl was added to each sample and centrifuged for 5 min at 16,249*g*. Two hundred and fifty microlitre supernatant was transferred to a new Eppendorf tube, taking care to avoid the precipitate. Five hundred microlitre ice‐cold 100% ethanol was added, before being cooled at −20**°**C overnight. The Eppendorfs were centrifuged at 16,249*g* for 30 min and the ethanol was tipped off. The DNA pellet was washed once with 1 ml 70% ethanol, before an additional 5 min in the centrifuge at 16,249 *g*. The DNA pellet was then dried at 50**°**C (*c*. 20 min), 200 μl molecular grade water added and the samples incubated at 37**°**C for 30 min. In addition to the stomach samples, negative controls, where nothing was dissected, but the tweezers were dipped in the digestion buffer at the beginning of the process, were included as contamination controls.

### 2.3 PCR and sequencing

The protocol developed previously by Lamb *et al.* (2017) was used here. In brief, the cnidarian‐specific *16s* mitochondrial (mt)DNA primers Scy_16s_f4 and Scy_16s_r4 were used to amplify a 135 bp amplicon in a PCR. The presence of a band at 177 bp on an ethidium‐bromide stained 1.5% agarose gel indicated cnidarians had been eaten. Positive PCR product was cleaned, using Exo1 (Thermo Scientific; http://www.thermoscientific.com) and FastAP (Thermo Scientific), then sanger‐sequenced (Eurofins UK; http://www.eurofins.co.uk). Sequences are included in Supporting Information Table S1. Identification of the consumed cnidarians based on basic local‐alignment search tool (BLAST)identity was performed using the nucleotide megablast algorithm (Altschul *et al.*, 1990) on the GenBank nucleotide database (Clark *et al.*, 2016).

### 2.4 Samples from 2008 and 2009

Additional DNA extracts from *Scomber scombrus* L. 1758 (*n* = 19) and *Sprattus sprattus* (L. 1758) (*n* = 609) stomachs, caught from the Irish Sea between 25 February and 2 March 2008 and 19–28 February 2009 in a previous study were also included. Stomachs were removed and frozen on‐board. At a molecular laboratory, *S. sprattus* stomachs had DNA extracted using the salt extraction technique as described here, *S. scombrus* stomachs had DNA extracted using a cetyltrimethylammonium bromide (CTAB) method (Fox *et al.*, 2012). PCR conditions were identical to those described here. Details of sampling approach, DNA extraction techniques and DNA sequences are available in full in Lamb *et al.* (2017).

### 2.5 Statistical analysis

A Fisher's exact test was performed to determine if differences in predation could be observed between seasons (February and March compared with October). Since multiple hypotheses (different species) were tested, a one‐stage false detection rate correction (Pike, 2011) was applied (reported as *q*‐values) to avoid the chance of a type‐2 error. All statistical analyses were performed using R (http://www.r-project.org).

## 3 RESULTS

### 3.1 Jellyfish predation

Cnidarian DNA was detected in three species: sardine *Sardina pilchardus* (Walbaum 1792), *S. scombrus* and *S. sprattus* (Table 1). Predation was rare in *S. pilchardus* and *S. sprattus* with only 3.3% and 1.4% samples containing jellyfish DNA in their stomachs respectively. Predation was common in *S. scombrus*, with 23.2% stomachs containing jellyfish DNA. The cnidarians consumed were identified as the scyphozoan *P. noctiluca*, as well as the hydrozoans *Geryonia proboscidalis* (Forsskål, 1775), *Scolionema suvaense* (Agassiz & Mayer, 1899) and *Liriope tetraphylla* (Chamisso & Eysenhardt, 1821) (no common names). Six *S. scombrus* and one *S. sprattus* could not be sequenced, these samples were excluded from the positive sample list. The successfully sequenced samples had BLAST identity values between 86% and 100% (Table 2).

**Table 2:** Species of jellyfish predators, the sampling station (Figure 1) and the jellyfish preyed upon that were detected using a 16s mtDNA assay

Species	Sampling station	Blast identification	
		Species	%
*Sardina pilchardus*	95	*Pelagia noctiluca*	100
*Scomber scombrus*	59	*Liriope tetraphylla*	94
*S. scombrus*	59	*P. noctiluca*	100
*S. scombrus*	59	*Geryonia proboscidalis*	86
*S. scombrus*	59	*L. tetraphylla*	99
*S. scombrus*	180	*L. tetraphylla*	97
*S. scombrus*	180	*L. tetraphylla*	97
*S. scombrus*	180	*L. tetraphylla*	97
*S. scombrus*	180	*L. tetraphylla*	93
*S. scombrus*	180	*L. tetraphylla*	95
*S. scombrus*	180	*L. tetraphylla*	100
*S. scombrus*	180	*L. tetraphylla*	100
*S. scombrus*	180	*L. tetraphylla*	96
*S. scombrus*	180	*L. tetraphylla*	99
*S. scombrus*	180	*L. tetraphylla*	100
*S. scombrus*	180	*L. tetraphylla*	99
*S. scombrus*	180	*L. tetraphylla*	100
*S. scombrus*	180	*L. tetraphylla*	100
*S. scombrus*	196	*L. tetraphylla*	90
*S. scombrus*	196	*Scolionema suvaense*	95
*S. scombrus*	196	*L. tetraphylla*	93
*S. scombrus*	196	*L. tetraphylla*	100
*S. scombrus*	196	*L. tetraphylla*	99
*Sprattus sprattus*	118	*P. noctiluca*	91
*S. sprattus*	118	*L. tetraphylla*	92
*S. sprattus*	118	*L. tetraphylla*	95

The BLAST identification shows the percentage of shared nucleotides with the sequence in the database and the length of the sequence used to identify the species.

### 3.2 Seasonal variation

Predation of jellyfish by *S. scombrus* was common in October (*n* = 22, 23.2% stomachs contained cnidarian DNA; late season), but was not detected in February or March (aggregated 2008 and 2009 data); a Fisher's exact test suggested this was a significant difference (*q* = 0.02, *P* = 0.01). Jellyfish appeared to be a rare prey item in both seasons (2015 data: *n* = 3, 1.4% stomachs contained jellyfish DNA; 2008–2009 data: *n* = 5, 0.8% stomachs contained jellyfish DNA) for *S. sprattus* and no significant difference was detected (*q* = 0.07, *P* > 0.05).

## 4 DISCUSSION

### 4.1 Observed predation

Three fish species: *S. sprattus*, *S. scombrus* and *S. pilchardus* were recorded as eating jellyfish. A single instance of *P. noctiluca* consumption was found in all three species. The ingestion of *G. proboscidalis* and *S. suvaense* was observed only once in *S. scombrus*, although it should be noted the low BLAST identification (86% and 95% respectively) suggests a high degree of uncertainty in the taxonomic assignment at the species or genus level: particularly as these species are not associated with the region. *L. tetraphylla* accounted for all remaining predation in *S. scombrus* and *S. sprattus*.

### 4.2 The pelagic food‐web

The most frequent consumer of jellyfish in the early season (February–March) was *C. harengus* (Lamb *et al.*, 2017), however none were captured in October and we were unable to draw comparison between early and late season. Seasonal comparisons in the other detected predators also presented challenges: in the early season a large sample size and a variety of trawling techniques were employed to capture both benthic and pelagic communities of fish (Fox *et al.*, 2012), facilitating the detection of rare predation. The late‐season samples were collected opportunistically through mid‐water trawling. Consequently, we were unable to capture the late‐season benthic component of the food web. Furthermore, the sample size was limited by a single‐individual processing these samples.

**Figure 2:**
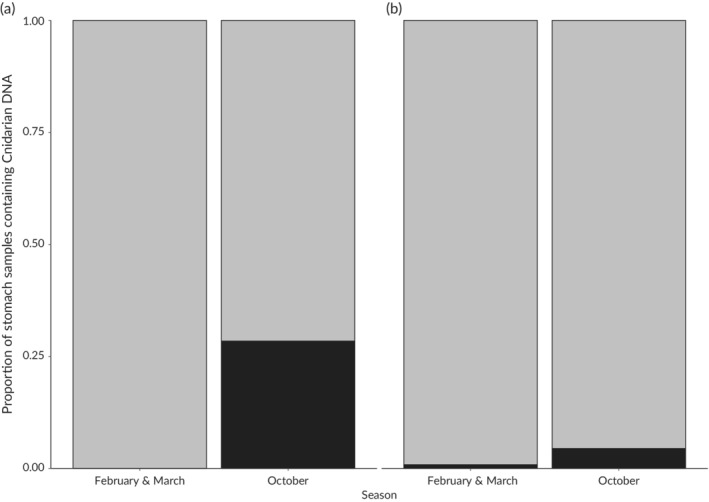
(a) Proportion of *Scomber scombrus* and (b) *Sprattus sprattus* stomachs in which jellyfish were detected in February–March 2008 and 2009, and October 2015. 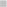, No cnidarians detected; 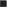, consumption of cnidarians had occurred

Although these factors limit our ability to assess temporal variation for the predatory fish species, these data still refine our understanding of jellyfish predation in the food‐web. Small jellyfish were a food source for *S. scombrus* (Figure 2): this has been recorded previously when *S. scombrus* switched from filter feeding to a biting in order to consume the small hydrozoan *Aglantha digitale* (O.F. Müller, 1776) (10–40 mm bell height; Runge *et al.*, 1987). However, in contrast to the widespread predation by fish during February and March (Lamb *et al.*, 2017) very little predation on jellyfish was found across the pelagic community during October. A possible explanation of diet shifts may be related to the relative abundance of other prey items. For example, *S. sprattus* switch to preying on fish eggs in the winter when other zooplankton levels are depressed (Pliru *et al.*, 2012). It is possible that widespread predation of scyphomeduase jellyfish ephyrae in the February and March is in response to poor availability of other zooplankton; greater zooplankton availability in October may result in a switch away from jellyfish and result in the observed predation rates.

### 4.3 Escaping predation?

**Figure 3:**
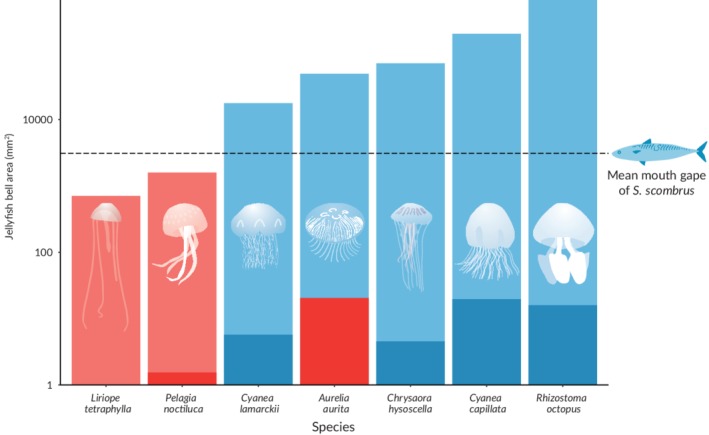
Jellyfish predation as a function of bell area (bar height). Predation of ephyrae is inferred using data from 2008–2009 (Lamb *et al.*, 2017), medusae predation was inferred using 2015 data presented in this paper. *n.b*. Bar graphs are overlaid, not stacked. Detection of predation (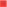) and non‐detection (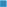) are shown for medusae (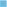, and 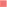) and ephyrae (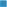 and 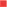). Mean *Lirope tetrayphylla* size and *Aurelia aurita* were taken from literature (Russell (1953) and Bastian *et al.* (2011), respectively). *Pelagia noctiluca* ephyrae size is taken from Sandrini & Avian (1983). All other mean medusae, and ephyrae, bell areas are reported in Holst (2012). The mouth gape of *Scomber scombrus* was calculated using mean *S. scombrus* size in this study and a *S. scombrus*‐specific allometric scaling function Karachle & Stergiou (2011)

Although no difference in seasonal predation was detected in *S. sprattus*, statistical analysis demonstrated *S. scombrus* fed on jellyfish more frequently in the samples collected in October than those in February and March. This was unexpected, as we anticipated the consumption of larger jellyfish to be more difficult and that rates of predation would therefore decline later in the year. Upon closer inspection however, the results do not contradict this hypothesis: *L. tetraphylla* has a bell diameter of 1–3 cm (Russell, 1953): 69 times smaller in area than a large *A. aurita* (bell diameter of 25 cm; Omori *et al.*, 1995). Larger jellyfish species such as *A. aurita*, *R. pulmo*, compass jellyfish *Chrysaora hysoscella* L. 1767 and blue jellyfish *Cyanea lamarcki* (Péron & Lesuer, 1810) were caught incidentally during the research cruise but were not detected with the dietary assay. While quantified population estimates are not available, this suggests that the complete absence of prey is unlikely to be responsible for jellyfishes’ absence in the dietary data. Prey switching could occur due to decreased medusae populations, which typically decrease and experience mortality, later in the year (Lucas, 2001), although overwintering populations have recently been recorded in other ecosystems (Ceh *et al.*, 2015; Purcell *et al.*, 2018). Another explanation is that larger species of jellyfish, particularly *A. aurita*, which were frequently preyed upon early in the season (which were likely ephyrae, although it should be noted this is inferred through phenological trends as the molecular techniques lack the ability to reveal this), may have escaped predation through somatic growth, leaving only small species like *L. tetraphylla* vulnerable to predation (Figure 3). Finally, it should be acknowledged that unknown sea‐specific phenomena may be driving the observed differences.

The data presented here show that, in contrast to early‐season sampling, late‐season predation is limited: *S. scombrus* were the only species to feed frequently on jellyfish, although some predation was also detected in *S. pilchardus* and *S. sprattus*. The type of jellyfish consumed also changed: the small hydrozoan species *L. tetraphylla* was the preferred prey item in October, accounting for 80.7% predation across all species. The shift from widespread predation of juvenile jellyfish to rare predation of adults suggests energy flows from jellyfish to fish stocks are dynamic throughout the year. Although jellyfish are not an energy‐rich food item when compared with other components of the plankton (Doyle *et al.*, 2007), the high abundance in which they can occur suggests they could play a role in supporting a range of forage‐fish populations during the winter. In late‐season sampling, consumption of jellyfish is less frequently seen. Possible explanations for this shift are changes in jellyfish availability, escape of predation through somatic growth, or sea‐specific phenomena. Collecting data on jellyfish populations throughout the year in one location, in tandem with diet‐sampling could elucidate which of these hypotheses, if any, are responsible for the observed predation patterns.

The jellyfish‐specific assay used here reveals the presence of a trophic link, but is not well suited to quantifying energy flows. Consequently, it is difficult to say exactly how important jellyfish are in the diet *S. scombrus*. Techniques such as stable‐isotope analysis could be used to quantify the energy flows between jellyfish and fish stocks. Additionally, high throughput sequencing with universal primers could reveal the broader context of diet: are jellyfish the only consumed prey or are they part of a generalist diet? Future research could use combination of both techniques to quantify jellyfish–fish trophic links.

## Supporting information


**TABLE S1** Sequences obtained from samples containing cnidarian *16s* mtDNA.
