# Supplementary material for: Cryptic diets of forage fish: jellyfish consumption observed in the Celtic Sea and western English Channel
Source: J Fish Biol. 2019 Mar 12;94(6):1026–32. doi: 10.1111/jfb.13926 (PMC6850654; doi:10.1111/jfb.13926)
Supplement: Supplementary file 1 — TABLE S1 Sequences obtained from samples containing cnidarian 16s mtDNA. [file JFB-94-1026-s001.rtf]

SUPPORTING INFORMATION

TABLE S1 Sequences obtained from samples containing cnidarian 16s mtDNA

Sardina pilchardus_9501_SCY_16S_F4
TTGGTGACTGGTATGAATGGTTAAACGAATATTCACCTGTCTCGGATAGAGACTTTATAAAATTGAATTTGCAGTGAAGATGCTGCGAGTGAATTGCA

Scomber scombrus_5902_SCY_16S_F4
CGTTTATTGACGACCACTATGAATGGTTAAACGAATTTTCCCCTGTCTCCCCGATAAACTTATTGAATTTAAAATAATAGTGAAGATGCTATTTATTAATTGT

S. scombrus_5910_SCY_16S_F4
ATTGGTGACTGGTATGAATGGTTAAACGAATATTCACCTGTCTCGGATAGAGACTTTATAAAATTGAATTTGCAGTGAAGATGCTGCGAGTGAATTG

S. scombrus_5912
TTGTCACTTTATTGACGACCGGTATGAATGGTTAAACGAATTTTCCCCTGTCTCCACGATAAGACTTGTATGAATTTGAATTAATAGTGAAGATGCTAGTCTTAATAATT

S. scombrus_5914
TGTCGTTTATTGACGACCAGTATGAATGGTTAAACGAATTTTCCACTGTCTCCACGATAAACTTAATGAATTTAAAATAATAGTGAAGATGCTATTTAATAATTGTA

S. scombrus_18002
TATGAATGGTTAAACGAATTTTCCACTGTCTCCNNNATAAACTTAATGAATTTAAAATAATAGTGAAGATGCTATTTAATAATTGTA

S. scombrus_18008
TTGTGGACAGGTATGAATGGTTAAACGAATTTTCCACTGTCTCCTCNATAAACTTAATGAATTTAAAATAATAGTGAAGATGCTATTTAATGATTGTA

S. scombrus_18009
GTATGAATGGTTAAACGAATTTTCCACTGTCTCCNCNATCAACTTAATGAATTTAAAATAATAGTGAAGATGCTATTTAATAATTGTA

S. scombrus_18010
TATGAATGGTTAAACGAATTTTCCACTGTCTNNNNNTAAACTTAATGAATTTAAAATAATAGTGAAGATGCTATTTAATAATTGTA

S. scombrus_18011
TATGAATGGTTAAACGAATTTTCCACTGTCTCNNNATAAACTTAATGAATTTAAAATAATAGTGAAGATGCTATTTAATAATTGTA

S. scombrus_18012
TTGACGACCAGTATGAATGGTTAAACGAATTTTCCACTGTCTCCACGATAAACTTAATGAATTTAAAATAATAGTGAAGATGCTATTTAATAATTGTA

S. scombrus_18013
GTATGAATGGTTAAACGAATTTTCCACTGTCTCCACGATAAACTTAATGAATTTAAAATAATAGTGAAGATGCTATTTAATAATTGT

S. scombrus_18014
ACCAGTATGAATGGTTAAACGAATTTTCCACTGTCTCNNNNATAAACTTAATGAATTTAAAATAATAGTGAAGATGCTATTTAATAATTGTA

S. scombrus_18015
CGTTTATTGACGACCAGTATGAATGGTTAAACGAATTTTCCACTGTCTCCACGATAAACTTAATGAATTTAAAATAATAGTGAAGATGCTATTTAATAATTGTA

S. scombrus_18016
TGACGACCAGTATGAATGGTTAAACGAATTTTCCACTGTCTCCACGATAAACTTAATGAATTTAAAATAATAGTGAAGATGCTATTTAATAATTGTA

S. scombrus_18017
ATGAATGGTTAAACGAATTTTCCACTGTCTCCACGACAAACTTAATGAATTTAAAATAATAGTGAAGATGCTATTTAATAATTGTA

S. scombrus_18021
TTGACGACCAGTATGAATGGTTAAACGAATTTTCCACTGTCTCCACGATAAACTTAATGAATTTAAAATAATAGTGAAGATGCTATTTAATAATTGTA

S. scombrus_18025
ATTGACGACCAGTATGAATGGTTAAACGAATTTTCCACTGTCTCCACGATAAACTTAATGAATTTAAAATAATAGTGAAGATGCTATTTAATAATTGTA

S. scombrus_19611
GACCAGTATGAATGGTTAAACGAATTTTTCACTGTCTCNNNCATAAACTTAATGAAATTGAAATTATAGTGAAGATGCTATTTTATAATTGTA

S. scombrus_19612
GATGACCAGTATGAATGGTTAAACGAATTTTTCACTGTCTCGATCATAAACCTAATGAAATTGTAATTATAGTGAAGATACTATTTTAAAATTGTA

S. scombrus_19617
GAACAGTATGAATGGTTAAACGAATTTTCCACTGTCTCCACNNTAAACTTAATGAATTTGAAATTGTAGTGAAGATGCTATTTAATAATTGCA

S. scombrus_19618
ATTGACGACCAGTATGAATGGTTAAACGAATTTTCCACTGTCTCCACGATAAACTTAATGAATTTAAAATAATAGTGAAGATGCTATTTAATAATTGTA

S. scombrus_19622
TATTGGAATGAGTTAAACGAATTTTCCACTGTCTCCACGATAAACTTAATGAATTTAAAATAATAGTGAAGATGCTATTTAATAATTGTA

Sprattus sprattus_11827
GACTGGTATGAATGGTTAAACGAATATTCACCTGTCTCNNNNNNNAGACNTATATAAAATTGAATTTGCAGTGAAGATGCTGCGAGTGAATTGCA

S. sprattus_11828
TATGAATGGTTAAACGAATTTTCCACTGTCTCNNNNNNAACTTAATGAATTTAAAATAATAGTGAAGATGCTATTTAATAATTGTA

S. sprattus_11830
CGTTTATTGACGACCAGTATGAATGGTTAAACGAATTTTCCACTGTCTCNNNNATAAACTTAATGAATTTAAAATAATAGTGAAGATGCTATTTAATAATTGTA
